# Supplementary material for: Third Early “Booster” Dose Strategy in France of bnt162b2 SARS-CoV-2 Vaccine in Allogeneic Hematopoietic Stem Cell Transplant Recipients Enhances Neutralizing Antibody Responses
Source: Viruses. 2022 Aug 30;14(9):1928. doi: 10.3390/v14091928 (PMC9506309; doi:10.3390/v14091928)
Supplement: Supplementary file 1 [file viruses-14-01928-s001.zip › viruses-1684913-supplementary.pdf]

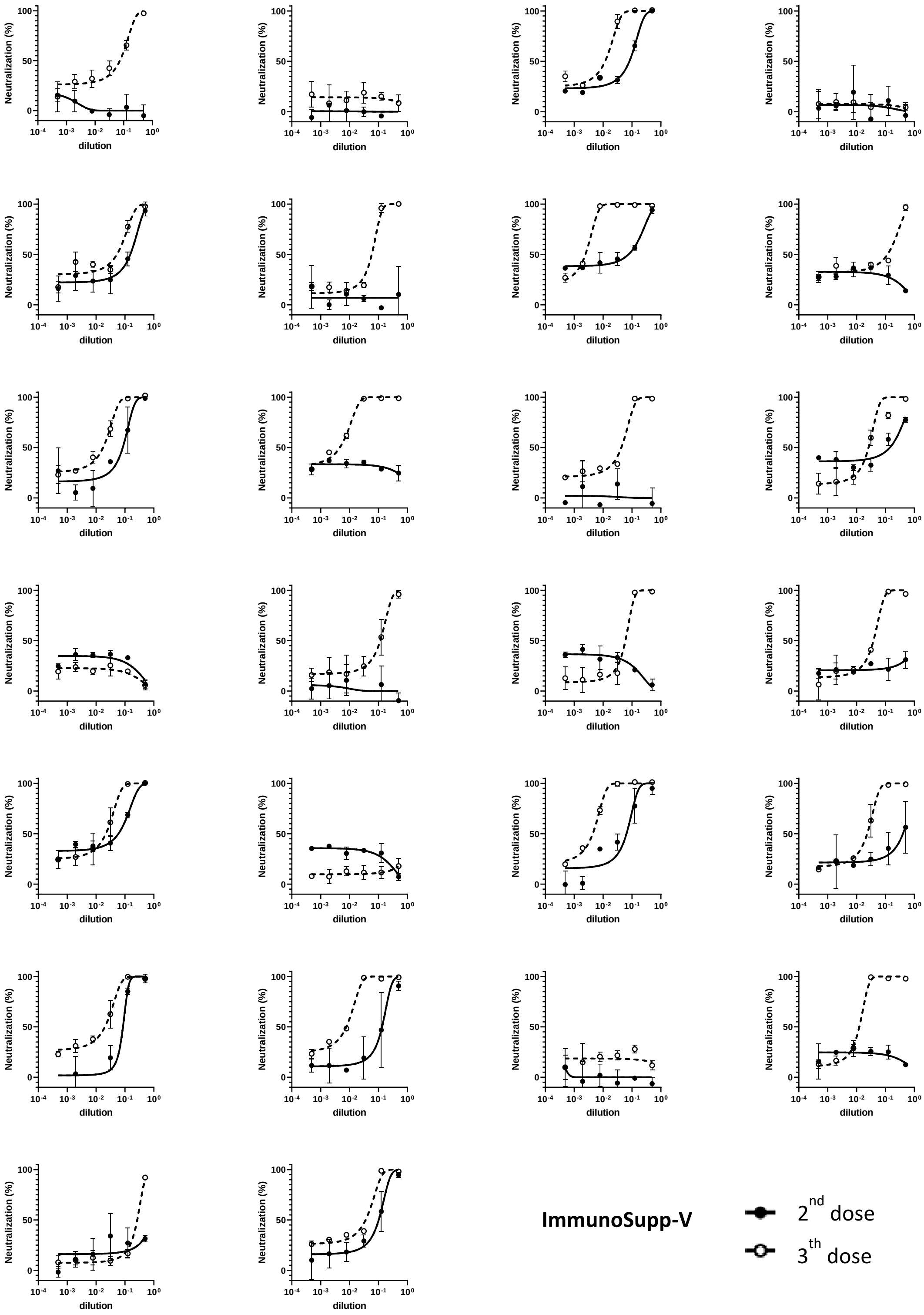

Figure S1. Individual neutralization curves against SARS-CoV-2 variant Delta of sera obtained after 2 doses (black line) or 3 doses (dotted line) of the BNT162b2 vaccine in 26 allogeneic HSCT recipients from the ImmunoSupp-V group. The experiments were performed in triplicate, and means±SD are shown.

Table S1. Amino acid substitutions in the Spike gene of the clinical samples and culture-expanded viruses. Viruses isolated from clinical samples and culture-expanded viruses were sequenced and their consensus genome sequences were compared to the reference sequence from Wuhan strain Hu-1 in bold (Accession number MN908947). Letters indicate amino acids, numbers indicate amino acid positions. NTD: N-terminal domain; RBD: receptor-binding domain; RBM: receptor-binding motif; FCS: furin cleavage site.

	Lineage		NTD						RBD						RBM	FCS				
		Spike position	18	69	70	241	242	243	417	452	477	478	484	501	614	681	682	683	684	685
		AA (Wuhan Hu-1)	**L**	**H**	**V**	**L**	**L**	**A**	**K**	**L**	**S**	**T**	**E**	**N**	**D**	**P**	**R**	**R**	**A**	**R**
D614G	B.1	Clinical sample													G					
		Expanded virus											D		G					H
Beta	B.1.351	Clinical sample	F			Δ	Δ	Δ	N				K	Y	G					
		Expanded virus	F			Δ	Δ	Δ	N				K	Y	G		Q			
Delta	B.1.617.2	Clinical sample								R		K			G	R				
		Expanded virus								R		K			G	R				
